# Up-Regulation of Adiponectin Expression in Antigravitational Soleus Muscle in Response to Unloading Followed by Reloading, and Functional Overloading in Mice

**DOI:** 10.1371/journal.pone.0081929

**Published:** 2013-12-06

**Authors:** Ayumi Goto, Yoshitaka Ohno, Akihiro Ikuta, Miho Suzuki, Tomotaka Ohira, Tatsuro Egawa, Takao Sugiura, Toshitada Yoshioka, Yoshinobu Ohira, Katsumasa Goto

**Affiliations:** 1 Department of Physiology, Graduate School of Health Sciences, Toyohashi SOZO University, Aichi, Japan; 2 Laboratory of Physiology, School of Health Sciences, Toyohashi SOZO University, Aichi, Japan; 3 Research Fellow of the Japan Society for the Promotion of Science, Tokyo, Japan; 4 Department of Exercise and Health Sciences, Yamaguchi University, Yamaguchi, Japan; 5 Hirosaki Gakuin University, Aomori, Japan; 6 Graduate School of Medicine, Osaka University, Osaka, Japan; University of Iowa, United States of America

## Abstract

The purpose of this study was to investigate the expression level of adiponectin and its related molecules in hypertrophied and atrophied skeletal muscle in mice. The expression was also evaluated in C2C12 myoblasts and myotubes. Both mRNA and protein expression of adiponectin, mRNA expression of adiponectin receptor (AdipoR) 1 and AdipoR2, and protein expression of adaptor protein containing pleckstrin homology domain, phosphotyrosine binding domain, and leucine zipper motif 1 (APPL1) were observed in C2C12 myoblasts. The expression levels of these molecules in myotubes were higher than those in myoblasts. The expression of adiponectin-related molecules in soleus muscle was observed at mRNA (adiponectin, AdipoR1, AdipoR2) and protein (adiponectin, APPL1) levels. The protein expression levels of adiponectin and APPL1 were up-regulated by 3 weeks of functional overloading. Down-regulation of AdipoR1 mRNA, but not AdipoR2 mRNA, was observed in atrophied soleus muscle. The expression of adiponectin protein, AdipoR1 mRNA, and APPL1 protein was up-regulated during regrowth of unloading-associated atrophied soleus muscle. Mechanical loading, which could increase skeletal muscle mass, might be a useful stimulus for the up-regulations of adiponectin and its related molecules in skeletal muscle.

## Introduction

Skeletal muscle demonstrates large plasticity in response to various extracellular stimuli. Numerous studies have demonstrated that mechanical loading, which is induced by resistance exercise as well as mechanical stretching, on skeletal muscle induces an increase in muscle mass, so-called muscle hypertrophy [1], [2], [3]. On the other hand, various disease- and trauma-associated long-term inactivity, immobilization and/or unloading are well known as major causes of skeletal muscle atrophy [4], [5], [6], [7]. These extracellular stimuli also cause changes in not only muscle mass but also the metabolic properties of skeletal muscles [8], [9].

Adiponectin, one of adipokines, has been intensively studied and is now known as a molecule which plays a crucial role in the regulation of insulin sensitivity [10], [11], [12]. It has been generally considered that adiponectin is synthesized and exclusively secreted in adipocytes. However, exercise-associated improvement of insulin sensitivity is not necessarily accompanied with increase in the level of adiponectin in circulation [13], [14], [15], [16]. Therefore, the origin of the adiponectin that acts on skeletal muscle cells, which are major target cells of adiponectin, remains unclear. Recent evidences have demonstrated the expression of adiponectin in non-adipocytes such as mouse C2C12 myotubes [17] and skeletal muscles [18], [19]. It has also been reported that differentiating C2C12 myotubes have an autocrine loop of adiponectin [20], suggesting that matured skeletal muscle cells may synthesize and secrete adiponectin in an autocrine manner.

Adiponectin enhances the β-oxidation of lipids and the utilization of glucose in skeletal muscle and its actions are mediated by binding to adiponectin receptors (AdipoRs), especially AdipoR1 [21]. Although two distinct AdipoRs, AdipoR1 and AdipoR2, have been cloned, AdipoR1 is predominant in skeletal muscle [22]. It has been reported that the up-regulation of AdipoR1 enhances local adiponectin sensitivity, and is sufficient to improve skeletal muscle insulin resistance [23]. However, there is no report regarding the expression levels of adiponectin, AdipoRs, especially AdipoR2, in hypertrophied and atrophied skeletal muscles.

Recently, an adaptor protein containing pleckstrin homology domain, phosphotyrosine binding domain, and leucine zipper motif 1 (APPL1) has been characterized as a mediating molecule for signaling downstream of AdipoR [24]. APPL1 interacts with components of the insulin signalling pathway such as phosphoinositide 3-kinase and phosphatidylinositol 3-kinase [25]. Overexpression of APPL1 increases fatty acid oxidation and glucose metabolism upon adiponectin stimulation [26]. However, the expression level of APPL1 in response to changes in skeletal muscle mass also remains unclear. Therefore, in the present study, we investigated the expression levels of adiponectin, AdipoRs, and APPL1 in hypertrophied and atrophied skeletal muscle in mice. The expression levels of adiponectin-related molecules in myoblasts and myotubes were also examined.

## Materials and Methods

### Cultured C2C12 cells and animals

Mouse myoblast cell-line C2C12 was used in the cell culture experiment. All animal protocols were carried out in accordance with the Guide for the Care and Use of Laboratory Animals as adopted and promulgated by the National Institutes of Health (Bethesda, MD) and were approved by the Animal Use Committee at Toyohashi SOZO University (A2010009, A2011001). All treatments of animals were performed under anesthesia with *i.p.* injection of sodium pentobarbital, and all efforts were made to prevent discomfort and suffering. Eleven-week-old male mice (C57BL/6J) were used. All mice were housed in a vivarium room with 12:12-h light:dark cycle and a maintained temperature and humidity of ∼23°C and ∼50%, respectively. Solid food and water were provided *ad libitum*.

### Cell culture experiments

Mouse C2C12 myoblasts were routinely cultured on culture plates with a genetic type I collagen bound surface (BD BioCoat, 6 wells, BD, NJ). Cells were grown in the growth medium consisting of Dulbecco's modified Eagle's medium (DMEM) supplemented with 10% fetal bovine serum containing high glucose (4,500 mg glucose/L, 4.0 mM L-glutamine, without sodium pyruvate) in a humidified atmosphere of 95% air and at 37°C in 5% CO2 until ∼80% confluent. The culture medium was then changed to a differentiation medium consisting of DMEM supplemented with 2% horse serum containing low glucose (1,000 mg glucose/L, 4.0 mM L-glutamine, 110 mg sodium pyruvate/L). Seven days after the exchanging of the medium, cells were collected as myotube samples for the analyses of mRNA and protein expressions. Approximately 90% of the cells had formed myotubes (data not shown). Some of the cells were collected myoblast samples immediately before the exchange of culture medium from growth to differentiation medium.

### Experiment of functional overloading

Functional overloading on the soleus of left hindlimb of mice (n = 10) was performed using the methods described previously [27]. Briefly, the distal tendons of plantaris and gastrocnemius muscle were cut under anesthesia with *i.p.* injection of sodium pentobarbital (50 mg/kg). Sham operation was performed, and the right soleus muscle was served as a control. 1 and 3 weeks after the treatment, the soleus muscles were dissected from both hindlimbs. The right soleus muscle was served as a contralateral control. Each muscle was cross-sectionally cut into two portions at the midbelly region. Proximal and distal portions were used for the analyses of protein and mRNA expressions, respectively.

### Experiment of hindlimb suspension and recovery

Twenty mice were randomly divided into 2 groups; 1) untreated pre-experimental control (n = 5) and 2) suspended groups (n = 15). Mice of the suspended group were subjected to continuous hindlimb suspension for 2 weeks. Hindlimb suspension was performed as described previously [28], [29]. Briefly, tails of the mice were cleaned, and were loosely surrounded by adhesive tapes cross-sectionally, fixing a string on the dorsal side of the tail, to keep the blood flow intact. The string was fastened to the roof of the cage at a height allowing the forelimbs to support the weight, yet preventing the hindlimbs from touching the floor and the sides of the cage. The mice could reach food and water freely by using their forelimbs. Immediately after 2 weeks of hindlimb suspension, ambulation recovery was allowed to mice (n = 10) in the suspended group.

The soleus muscles of the suspended group were dissected from bilaterally before and immediately, 2, and 4 weeks after 2 weeks of the suspension under anesthesia with *i.p.* injection of sodium pentobarbital (50 mg/kg). The muscles were trimmed of excess fat and connective tissues, weighed, frozen in liquid nitrogen, and stored at −80°C. The left and the right muscles were used for the analysis of protein expression and also for the analysis of mRNA expression.

### Real-time Reverse Transcription-PCR

For mRNA analyses, cells and muscle tissues were incubated in RNAlater® (Qiagen GmbH, Hiden, Germany) and stored at −80°C until the extraction of total RNA. Total RNA was extracted from C2C12 cells and soleus muscles using the RNeasy Mini Kit (Qiagen) according to the manufacture's protocol. Samples (∼40 ng RNA) were reverse-transcribed into complementary DNA (cDNA) by using the first-standard cDNA Synthesis kit according to the manufacture's instructions [PrimeScript RT Master Mix (Perfect Real Time) for mRNA, Takara Bio, Otsu, Japan]. Synthesized cDNA was applied to real-time reverse transcription-PCR (Thermal Cycler Dice® Real Time System II MRQ, Takara Bio) using Takara SYBR *Premix Ex Taq* II for mRNA, and analyzed with Takara Thermal Cycler Dice® Real Time System Software Ver. 4.00 according to the manufacturer's instructions. The real-time cycle conditions were 95°C for 30 s followed by 40 cycles at 95°C for 5 s and at 60°C for 30 s for mRNA. Relative gene expression was quantified by the 2^−ΔΔCT^ method. The quality of extracted RNA was evaluated by O.D. 260/280 ratio. In the present study, the values of all samples were more than 1.9. Specificity was confirmed by electrophoretic analysis of the reaction products and by inclusion of template- or reverse transcriptase-free controls. To normalize the amount of total RNA present in each reaction, we determined the best internal control for mRNA expressions by using NormFinder that is an algorithm for identifying the optimal normalization gene among a set of candidates [30], glyceraldehyde-3-phosphase dehydrogenase (GAPDH), β-actin, and S18 ribosomal RNA (18 S rRNA). Judging from these comparisons (Figs S1–S3), GAPDH was the most stable mRNA. Therefore, GAPDH mRNA was used for an internal control for mRNA expressions in each experiment.

The primers were designed by using the Takara Bio Perfect Real Time Support System (Takara Bio). Primers used for detection mouse cDNA were as follows: adiponectin, 3′-GTCAGTGGATCTGACGACACCAA-5′ (forward) and 3′-ATGCCTGCCATCCAACCTG-5′ (reverse); AdipoR1, 3′-CTGGGCATCTCTGCCATCA-5′ (forward) and 3′-CTTGACAAAGCCCTCAGCGATA-5′ (reverse); AdipoR2, 3′-ATCAGCAGCCAGACGCACTC-5′ (forward) and 3′-TGACCAGTCCCAAAGACCTCTACTC-5′ (reverse); GAPDH, 3′-TGTGTCCGTCGTGGATCTGA-5′ (forward) and 3′-TTGCTGTTGAAGTCGCAGGAG-5′ (reverse); β-actin, 3′- CATCCGTAAAGACCTCTATGCCAAC-5′ (forward) and 3′- ATGGAGCCACCGATCCACA-5′ (reverse) and 18 S rRNA, 3′-ACTCAACACGGGAAACCTCA-5′ (forward) and 3′- AACCAGACAAATCGCTCCAC-5′ (reverse).

### Immunoblotting analyses

For the analyses of protein expressions, collected cells and muscles were homogenized in an isolation buffer (CelLytic MT, Sigma-Aldrich, St. Louis, MO) with 1 mM Na_3_VO_4_, 1 mM phenylmethylsulfonyl fluoride (PMSF) and 1 µg/ml leupeptin with glass homogenizer. The homogenates were then sonicated and centrifuged at 12,000 rpm (4°C for 10 min), the supernatant was collected. A part of the supernatant was solubilized in sodium-dodecyl sulfate (SDS) sample buffer {30% (vol/vol) glycerol, 5% (vol/vol) 2-mercaptoethanol, 2.3% (wt/vol) SDS, 62.5 mM Tris·HCl, 0.05% (wt/vol) bromophenol blue, and pH 6.8} at a concentration of 0.5 mg of protein per milliliter and boiled for 5 min. The SDS-polyacrylamide gel electrophoresis (SDS-PAGE) was carried out on 10% polyacrylamide [bisacrylamide/acrylamide, 1∶20 (wt/wt)] slab gel (60×85×1 mm) containing 0.5% SDS at a constant current of 20 mA for 120 min. Equal amounts of protein (10 µg) were loaded on each gel. Molecular weight markers (ECL DualVue Western Blotting Markers, GE Healthcare, Buckinghamshire, UK) were applied to both sides of 14 lanes as the internal controls for transfer process or electrophoresis.

Following SDS-PAGE, proteins were transferred to polyvinylidene difluoride (PVDF) membranes (0.2 µm pore size, Bio-Rad) at a constant voltage of 100 V for 60 min at 4°C. The membranes were blocked for 1 h using a blocking buffer: 5% skim milk with 0.1% Tween 20 in Tris-buffered saline (TTBS) with pH 7.5. The membranes were incubated for overnight at 4°C with a polyclonal antibody for adiponectin (R&D Systems, Minneapolis, MN), APPL1 (Cell Signaling Technology, Beverly, MA), GAPDH (Sigma-Aldrich), and β-actin (Sigma-Aldrich) and then reacted with a secondary antibody conjugated to horseradish peroxidase (anti-rabbit IgG: Cell Signaling Technology, anti-goat IgG: Jackson ImmunoResearch Laboratories, West Grove, PA) for 1 or 2 h. After the final wash, protein bands were visualized using chemiluminescence (ECL Advance Western blotting kit, GE Healthcare), and signal density was measured using Light-Capture (AE-6971) with CS Analyzer Ver. 2.08b (ATTO corporation, Tokyo, Japan).

In the present study, we investigated which was the best internal control for protein expression, GAPDH or β-actin (Figs. S4–S6). As the most stable protein expression of GAPDH per a unit of muscular soluble protein was observed in the cell culture and the functional overloading experiment, the protein expression level of GAPDH in each sample of these experiments was used for an internal control. The protein expression levels of adiponectin and APPL1 from these experiments were normalized by using the expression level of GAPDH protein. On the other hand, in the hindlimb suspension and recovery experiment, the protein expression levels of adiponectin and APPL1 were normalized using the protein expression level of β-actin. Each sample was investigated in, at least, duplicate to ensure that results were not influenced by loading errors.

### Statistical analyses

All values were expressed as means ± SEM. The statistical significance of the values from the cell culture experiments was analyzed by F-test followed by unpaired Student's t-test. For the statistical analyses of the values from the experiments of functional overloading and hindlimb suspension and recovery, the normal distribution of the data was evaluated by using Kolmogorov-Smirnov test. Statistical significance of the values from the functional overloading experiment was analyzed by using 2-way (treatment x time) analysis of variance (ANOVA) followed by Turkey-Kramer post hoc test (relative muscle wet weight and AdipoR1 mRNA) or, if normality failed, by Kruskal-Wallis test followed by Steel-Dwass test (adiponectin mRNA and protein, AdipoR2 mRNA, and APPL1 protein). Since all data from the experiment in which hind-limb suspension followed by ambulation recovery was carried out, was confirmed to fit the normal distribution, the statistical significance of the values was analyzed by using one-way ANOVA followed by Turkey-Kramer post hoc test. Spearman's rank correlation coefficients were calculated to assess the interrelationships between the relative muscle wet weight and the protein expression levels of adiponectin or APPL1, or the mRNA expression levels of AdipoR1 and AdipoR2. The significance level was accepted at p<0.05.

## Results

### Myoblasts and myotubes

**Figure 1:**
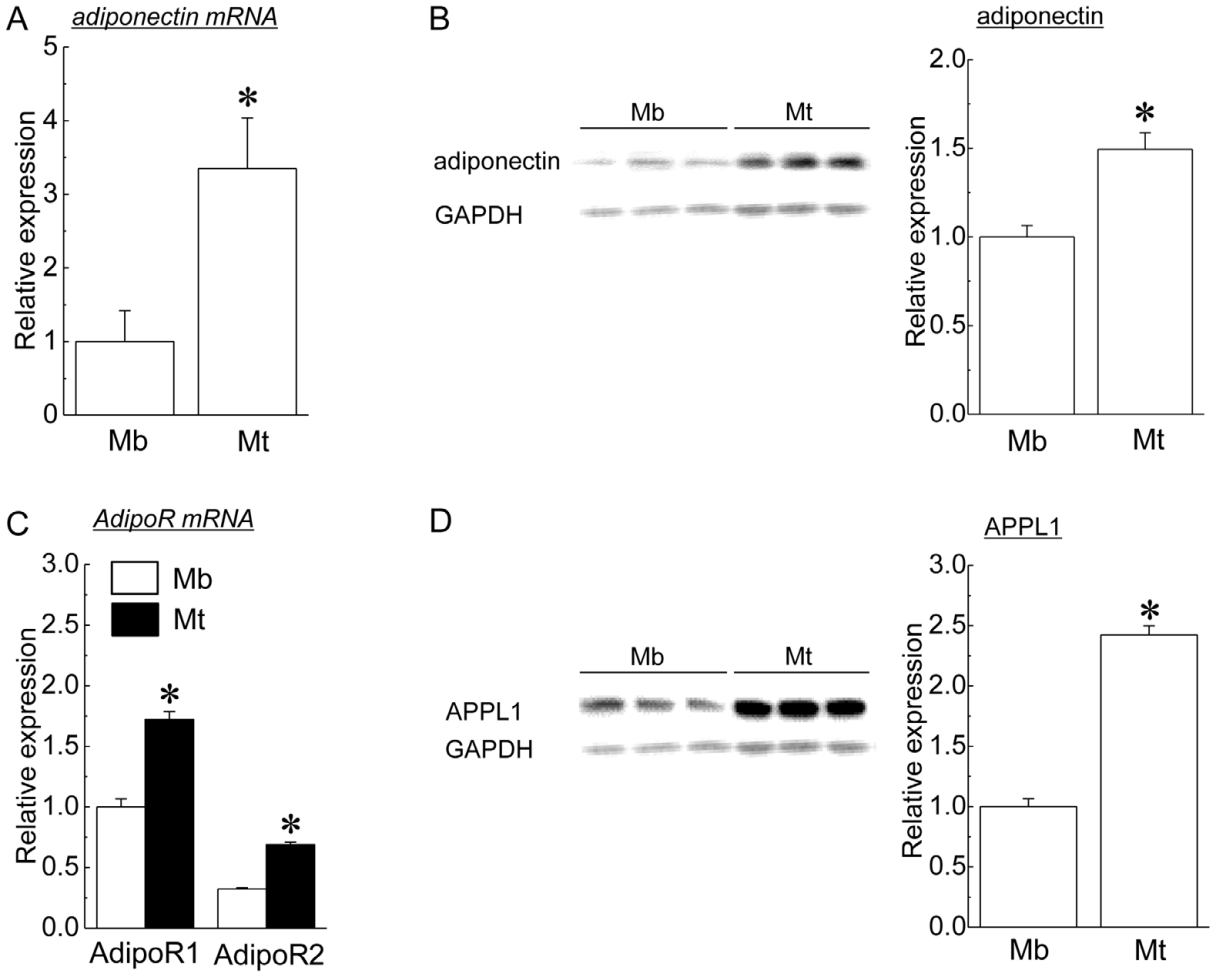
Expression levels of adiponectin and its related molecules in myoblasts (Mb) and myotubes (Mt). A: adiponectin mRNA, B: adiponectin protein, C: adiponectin receptor 1 (AdipoR1) mRNA and adiponectin receptor 2 (AdipoR2) mRNA, D: adaptor protein containing pleckstrin homology domain, phosphotyrosine binding domain, and leucine zipper motif 1 (APPL1) protein. The protein expression levels of adiponectin and APPL1 were shown as relative values to the expression level of glyceraldehyde-3-phosphate dehydrogenase (GAPDH) protein. Mean ± SEM. n = 6. *: significantly different from the value of myoblasts (Mb) (p<0.05).

In the present study, we compared the expression levels of adiponectin and its related molecules between C2C12 myoblasts and myotubes. Differentiation of myoblasts to myotubes was judged by using the microscopic images of the cells (Fig. S7). Figure 1 shows the mean expression levels of adiponectin and its related molecules in myoblasts and myotubes of C2C12. Both mRNA and protein expressions of adiponectin were observed in not only myotubes but also myoblasts (Figs. 1A and 1B). Expression of adiponectin mRNA and protein in myotubes was significantly higher than for those in myoblasts (p<0.05). In addition, mRNA expression of AdipoR1 and AdipoR2 was also observed in both myoblasts and myotubes (Fig. 1C). The mRNA expression levels of both AdipoR1 and AdipoR2 in myotubes were also significantly higher than for those in myoblasts (p<0.05). The expression of APPL1 protein was observed in both myoblasts and myotubes. The mean expression level of APPL1 protein in myotubes was significantly higher than that in myoblasts (p<0.05, Fig. 1D).

### Effects of functional overloading

**Figure 2:**
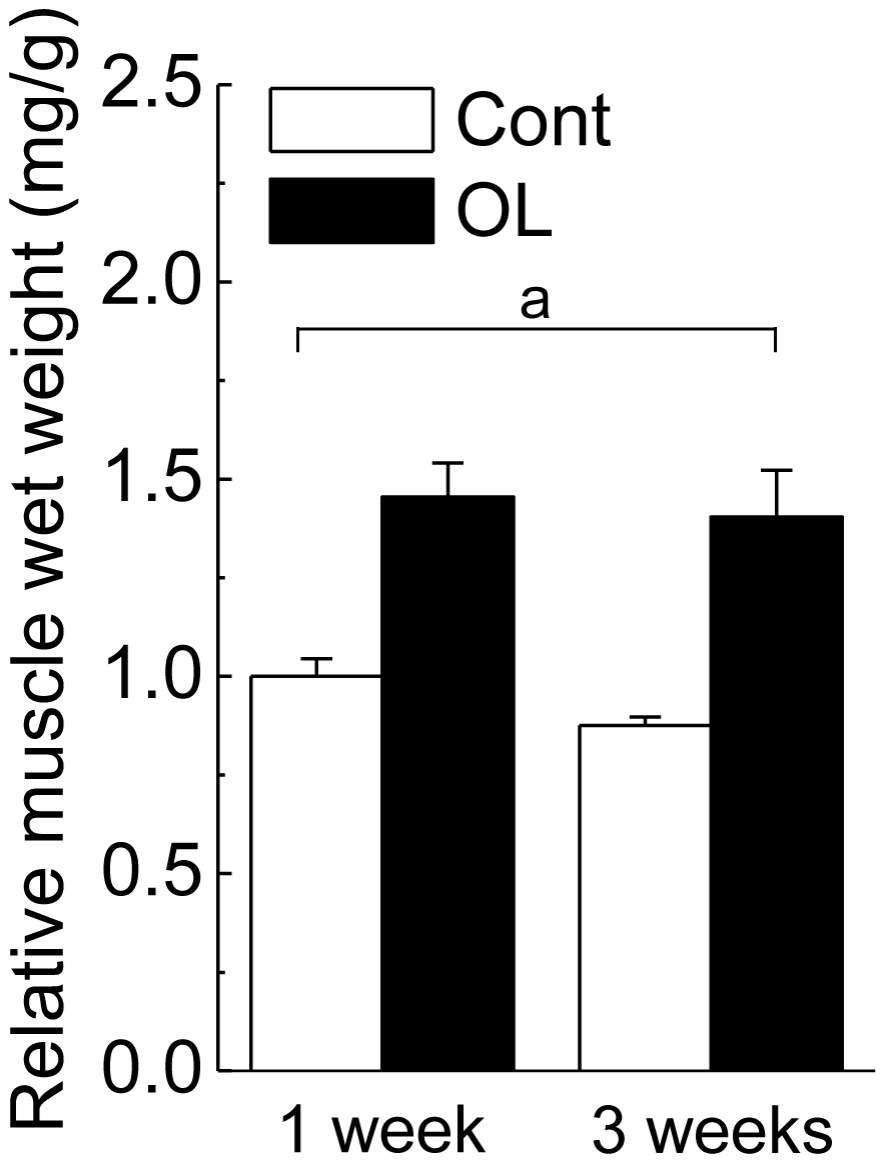
Changes in soleus muscle wet weight in response to functional overloading. The values show the muscle wet weight relative to body weight. Cont: untreated control group, OL: functional overloaded group, 1 week and 3 weeks: 1- and 3-week of functional overloading. Mean ± SEM. n = 5/group at each time point. pone.0081929.a.tif: a significant effect of treatment (p<0.05) revealed by two-way ANOVA (treatment and time).

The wet weights of soleus muscle relative to body weight in both untreated contralateral control and overloaded groups are shown in Fig. 2. Two-way ANOVA (treatment x time) for the changes in the relative soleus weight showed a significant effect of treatment (p<0.05), but not of time. Muscle wet weight was increased by ∼1.45-fold following 1 and 3 weeks of overloading, compared with the values of contralateral control.

**Figure 3:**
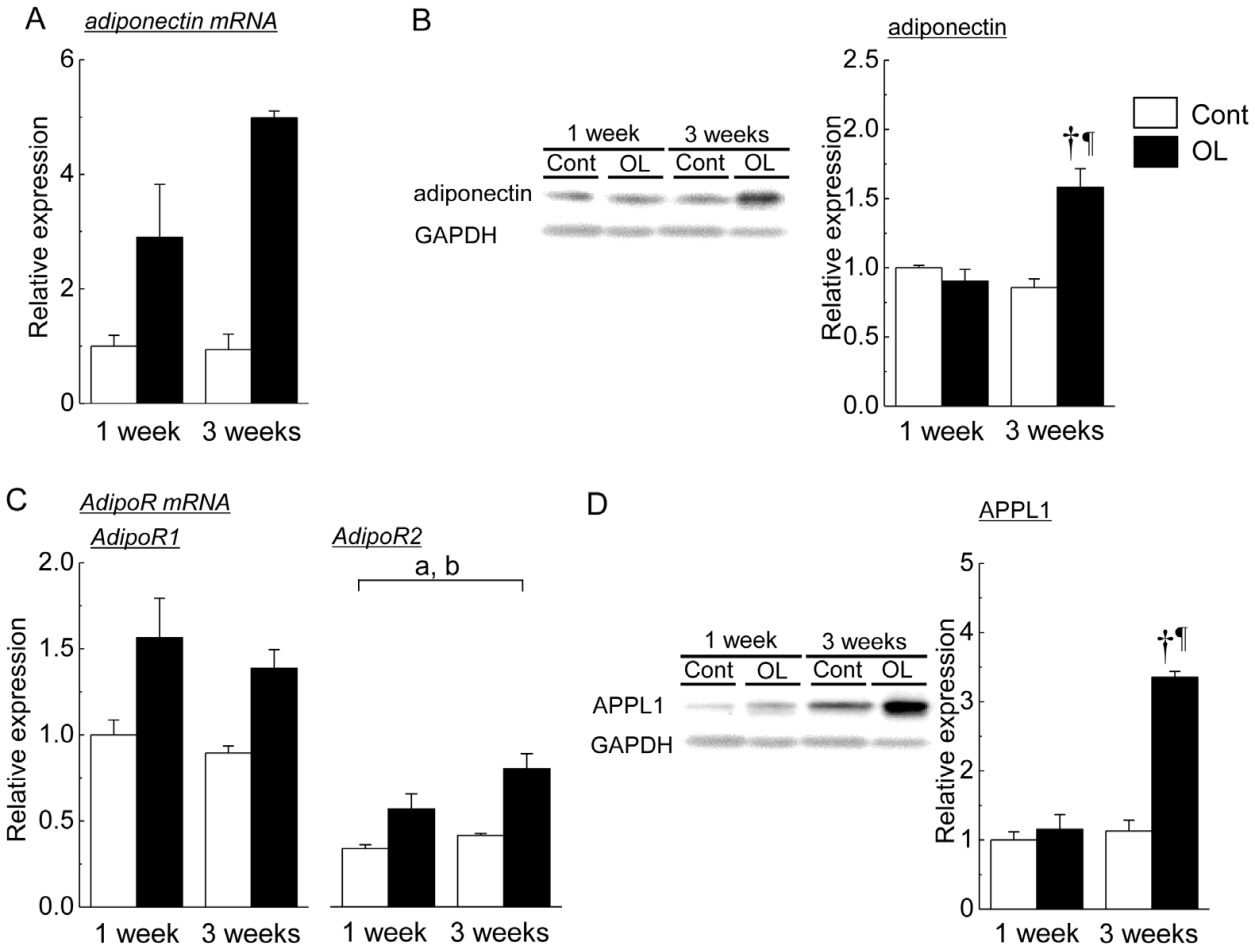
Expression levels of adiponectin and its related molecules in soleus muscle in response to functional overloading. A: adiponectin mRNA, B: adiponectin protein, C: adiponectin receptor 1 (AdipoR1) mRNA and adiponectin receptor 2 (AdipoR2) mRNA, D: adaptor protein containing pleckstrin homology domain, phosphotyrosine binding domain, and leucine zipper motif 1 (APPL1) protein. The protein expression levels of adiponectin and APPL1 were shown as the relative values to the expression level of glyceraldehyde-3-phosphate dehydrogenase (GAPDH) protein. Mean ± SEM. n = 5. Cont: untreated control group, overloaded: functional overloaded group. See figures 1 and 2 for other abbreviations. a and b: a significant effect of treatment (p<0.05) and a significant effect of time revealed by two-way ANOVA (treatment and time). *, †, and **¶**: p<0.05 vs. 1 week of control group, 1 week of overloaded group, and 3 weeks of control group, respectively.

Figure 3 shows the changes in adiponectin and its related molecules in the soleus muscle of both the contralateral control and overloaded soleus muscles. Although there was no significant difference in mRNA expression of adiponectin (Fig. 3A), the protein expression level was significantly increased following 3 weeks of functional overloading, compared with the levels of contralateral control and following 1 week of overloading (Fig. 3 B, p<0.05). Two-way ANOVA revealed that significant effects of treatment and time in changes in mRNA expression levels of AdipoR2 were observed (p<0.05, Fig. 3C). Figure 3D shows the changes in the protein expression level of APPL1 in the soleus muscle of both groups. A significant up-regulation of APPL1 protein expression was observed following 3 weeks of functional overloading, compared with the levels of contralateral control and following 1 week of overloading (p<0.05).

### Effects of unloading and reloading

**Figure 4:**
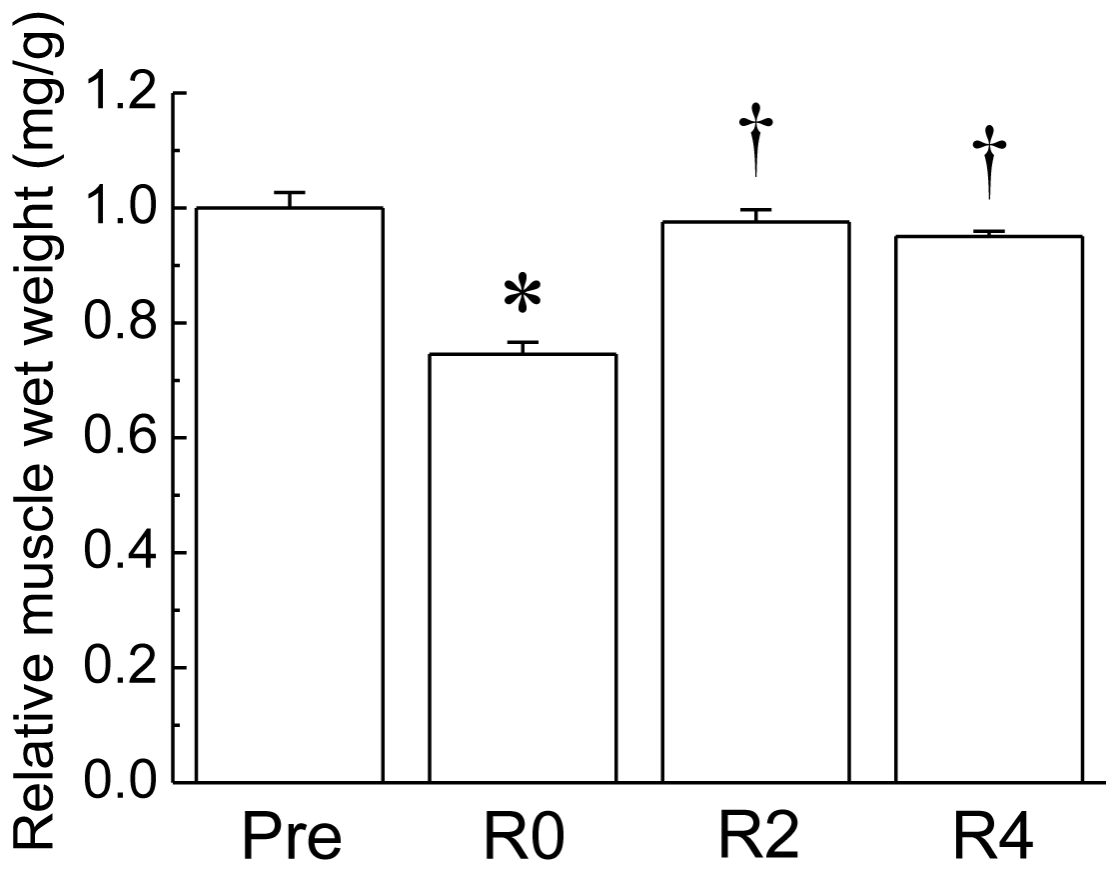
Muscle wet weight in response to 2 weeks of hindlimb suspension followed by 4 weeks of recovery. Pre: before hindlimb suspension. R0, R2, and R2: immediately, 2 and 4 weeks of respectively recovery period after the suspension. The values show the muscle wet weight relative to body weight. Mean ± SEM. n = 5. * and †: p<0.05 vs. Pre and R0, respectively.

Changes in the relative soleus weight during 2-week of hindlimb suspension followed by 4-week of ambulation recovery are shown in Fig. 4. Immediately after 2 weeks of the suspension (R0), the relative soleus weight was significantly decreased (p<0.05). The relative weights at 2 (R2) and 4 weeks (R4) of recovery after the suspension were significantly higher than that at R0 (p<0.05).

**Figure 5:**
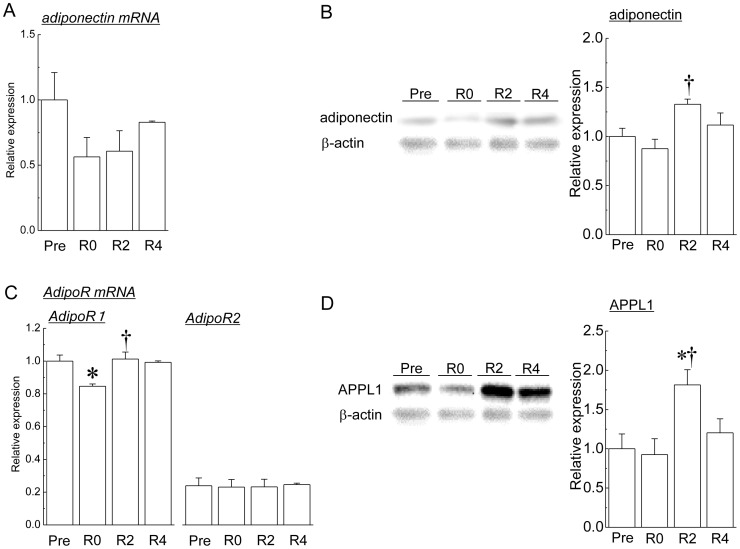
Expression levels of adiponectin and its molecules in muscle in response to hindlimb unloading followed by reloading. A: adiponectin mRNA, B: adiponectin protein, C: adiponectin receptor 1 (AdipoR1) mRNA and adiponectin receptor 2 (AdipoR2) mRNA, D: adaptor protein containing pleckstrin homology domain, phosphotyrosine binding domain, and leucine zipper motif 1 (APPL1) protein. The protein expression levels of adiponectin and APPL1 were shown as the relative values to the expression level of β-actin protein. Mean ± SEM. n = 5. See figures 1, 2, and 3 for abbreviations. * and †: p<0.05 vs. Pre and R0, respectively.

Figure 5 shows the changes in adiponectin and its related molecules in soleus muscle during the suspension followed by the recovery period. Although there were no significant differences in the mRNA expression levels of adiponectin during the experimental period (Fig. 5A), the protein expression level of adiponectin tended to be decreased by 2 weeks of the suspension, and was significantly increased by 2 weeks of the recovery (p<0.05, Fig. 5B). The mean expression level of AdipoR1 mRNA was significantly decreased by 2 weeks of the suspension, and was significantly increased during 2 weeks of the recovery (p<0.05, Fig. 5C). Conversely, no significant changes were observed in the expression level of AdipoR2 mRNA during the suspension and the recovery (Fig. 5C). Significant increase in the mean expression of APPL1 protein was observed 2 weeks after the recovery, compared with the expression levels before and immediately after the suspension (p<0.05, Fig. 5D).

### Interrelationship between muscle wet weight and adiponectin, AdipoRs or APPL1

**Figure 6:**
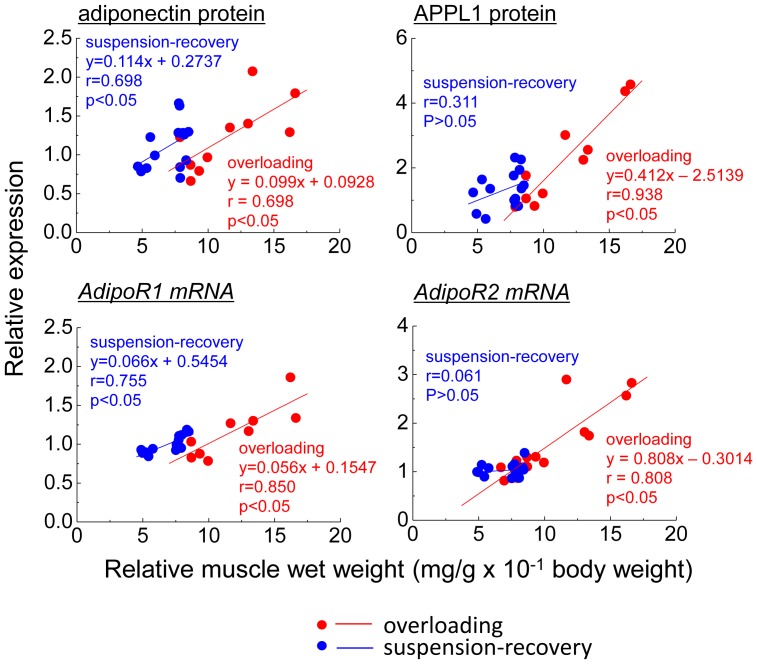
Interrelationship between soleus muscle wet weight relative to body weight and the expression levels of the expression levels of adiponectin protein, APPL1 protein, AdipoR1 mRNA or AdipoR2 mRNA. Red circle: data from the experiment of functional overloading experiment (contralateral control and following 3 weeks of functional overloading). Blue circle: data from the experiment of hindlimb suspension and recovery (before, 2 weeks of the suspension, and 2 and 4 weeks of recovery following the suspension). A linear regression line was calculated by using of data each experiment in each graph.

Figure 6 shows the interrelationships between soleus muscle wet weight relative to body weight and the expression levels of adiponectin-related molecules. The values were plotted from the contralateral control and 3 weeks of functional overloading in the functional overloading experiment both before and immediately, 2, and 4 weeks respectively after 2 weeks of the suspension in the suspension-recovery experiment, since there was no significant increase in the relative soleus muscle weight following 1 week of the overloading. In the overloading experiment, there were positive interrelationships between the soleus muscle wet weight relative to body weight and the expression levels of adiponectin protein, APPL1 protein, AdipoR1 mRNA, or AdipoR2 mRNA (p<0.05). Positive relation between the muscle weight and the expression levels of adiponectin protein or AdipoR1 mRNA were also observed in the suspension-recovery experiment (p<0.05). However, there was no significant relationship between soleus muscle weight and APPL1 protein or AdipoR2 mRNA in the suspension-recovery experiment.

## Discussion

The present study showed both mRNA and protein expressions of adiponectin, mRNA expressions of AdipoR1 and AdipoR2, and protein expression of APPL1 in C2C12 myoblasts. In C2C12 myotubes, the expression levels of adiponectin-related molecules were higher than those in myoblasts. The expression of adiponectin-related molecules in antigravitational soleus muscle was observed at mRNA (adiponectin, AdipoR1, AdipoR2) and protein (adiponectin, APPL1) levels. The protein expression levels of adiponectin and APPL1 in soleus muscle were up-regulated following 3 weeks of functional overloading. On the other hand, down-regulation of AdipoR1 mRNA, but not AdipoR2 mRNA, was observed in atrophied soleus muscle. The expression levels of adiponectin protein, AdipoR1 mRNA, and APPL1 protein were up-regulated during the regrowth of unloading-associated atrophied soleus muscle. There were significant positive interrelationships between the muscle wet weight and the expression levels of adiponectin-related molecules in overloading-associated hypertrophied skeletal muscle. However, there were significant interrelationships between the muscle wet weight and the expression levels of adiponectin protein or AdipoR1 mRNA, but not APPL1 protein and AdipoR2 mRNA, in atrophied and regrowing skeletal muscle.

### Myogenic differentiation and adiponectin expression

In the present study, the expression of adiponectin and its related molecules was observed in both myoblasts and myotubes of cultured C2C12 cells. Although the expression of AdipoR1, AdipoR2, and APPL1 in myoblasts was also confirmed in the previous study [20], [31], [32], this is the first study showing the mRNA and protein expression of adiponectin in C2C12 myoblasts. A previous study [20] has reported that the protein expression of adiponectin had been observed in C2C12 myotubes, but not in myoblasts. Although we have no clear explanation for the discrepancy between the results obtained in the previous investigation [20] and the present study, the mRNA expression of adiponectin in myoblasts may support our result that myoblasts have endogenous expression of adiponectin protein.

We also found that the up-regulation of adiponectin and its related molecules was observed in myotubes, rather than myoblasts. Up-regulation of APPL1 protein during myogenic differentiation was consistent with the results from the previous study [32]. However, it has been reported that the mRNA expression levels of AdipoR1 and AdipoR2 were constant during the differentiation [20], but this result was inconsistent with the present study. In the previous study, however, the mRNA expression levels of AdipoRs were evaluated using 3-day-differentiating myotubes [20]. On the other hand, we investigated the mRNA expression levels of 7-day-differentiating myotubes. Since the number of undifferentiated C2C12 cells on the ∼4^th^ day of the differentiation were higher than that on the 7^th^ day of the differentiation (Fig. S7), the discrepancy in the changes in AdipoR mRNAs expression between the results of the previous study [20] and the present study may be attributed to the differentiation stages of C2C12 myotubes.

### Overloading and unloading followed by reloading

In the present study, soleus muscle wet weight was increased by ∼1.45-fold following 3 weeks of overloading, compared with the values of contralateral control. A similar increment of overloading-associated soleus muscle was observed in the previous studies [27], [33], [34]. Protein expression levels of adiponectin in skeletal muscle were up-regulated by 3 weeks of functional overloading. In addition, the up-regulation of adiponectin in skeletal muscle was also observed by reloading on unloading-associated atrophied muscle. This is the first study showing the expression levels of adiponectin in skeletal muscle in response to overloading and unloading followed by reloading.

It has been suggested that some of the overloading- and reloading-associated increase in antigravitational soleus muscle wet weight may be attributed to some damage, inflammation and swelling in the loaded muscle during the early stage of adaptation (∼5–7 days of overloading and ∼4–5 days of reloading, respectively) [35], [36], [37]. However, it is unlikely that the increase of soleus muscle in the present study (except for 1 week of the overloading) is affected by such acute affects because the muscle was sampled after 3 weeks of overloading, and after 2 and 4 weeks of reloading following unloading. In addition, the previous study reported that muscle protein content increased following 3 weeks of overloading [27] and 2 weeks of reloading following 2 weeks of hindlimb suspension [38].

In the present study, up-regulation of AdipoR1 mRNA was observed in reloaded soleus muscle. Up-regulation of AdipoR2 was also observed in overloaded muscle. On the other hand, the down-regulation of AdipoR1 mRNA, but not AdipoR2 mRNA, was observed in atrophied soleus muscle. Several reports have shown that AdipoR1 mRNA level in skeletal muscle was up-regulated by running exercise in rats [39] and mice [40], and by cycling, running or swimming exercise in healthy and type 2 diabetes human [16]. Since stress proteins, namely heat shock proteins, in skeletal muscle are up-regulated in functionally overloading [41] as well as reloading following hindlimb unloading [29], AdipoR1 in skeletal muscle may be up-regulated by loading-related cellular stress.

It has been reported that the expression level of AdipoR1in skeletal muscle correlates with glucose and lipid metabolism, and insulin sensitivity [42], [43], [44]. Although insulin sensitivity was not evaluated in the present study, loading-associated up-regulation of AdipoR1 in skeletal muscle could improve insulin sensitivity. However, a physiological significance of AdipoR2 expression in response to overloading and reloading following unloading remains unclear.

In the present study, APPL1 protein was also up-regulated in overloaded and reloaded soleus muscle following unloading. This is the first study showing the responses of APPL1 expression in skeletal muscle in response to overloading, unloading, and reloading. Although we have no clear explanation regarding the up-regulation of APPL1in hypertrophied and reloaded skeletal muscle, the up-regulation of APPL1 may be associated with the up-regulations of adiponectin and AdipoRs in skeletal muscle. Collectively, all the aforementioned results suggest that the expressions of adiponectin and its related molecules including APPL1 could be up-regulated by increasing load on skeletal muscle, suggesting that these phenomena could enhance adiponectin action.

Numerous studies have shown the transition of type II to type I fibers in overloading-associated soleus muscle hypertrophy in mice [34] and in rats [45], [46]. In addition, unloading-associated decrease in type I myosin heavy chain (MHC) [47], [48], [49], [50] and reloading-associated recovery of type I MHC [48], [51], [52] in rats and mice are also well known. Muscle hypertrophy- and atrophy-associated transition of fiber types may influence the expression levels of adiponectin-related molecules since type IIA and IID fibers contain high adiponectin in comparison to type IIB and type I fibers [18]. In the present study, the expression levels of adiponectin-related molecules were up-regulated in overloading-associated hypertrophied mouse soleus muscle. On the contrary, protein expression level of adiponectin tended to be decreased by 2 weeks of the suspension, and was significantly increased by 2 weeks of the recovery (Fig. 5). Although we did not investigate the changes in fiber type in soleus muscle following overloading, hindlimb unloading, and reloading, it is unlikely that a shift in fiber type distribution affected adiponectin regulation in soleus muscle.

In the present study, there were positive interrelationships between soleus muscle wet weight relative to body weight and the expression levels of adiponectin protein or AdipoR1 mRNA in the experiments of functional overloading and hindlimb suspension-recovery experiments, suggesting that muscle mass might implicate adiponectin content. However, positive relationships between the muscle weight and APPL1 protein or AdipoR2 mRNA were observed in the overloading experiment, but not in the suspension-recovery experiment. Therefore, there is a possibility that the regulation mechanism(s) of adiponectin-related molecules expressions during hypertrophy of normal skeletal muscle might be different from that during the regrowth of atrophied skeletal muscle. Additional studies will be needed to elucidate this issue.

In conclusion, both mRNA and protein expressions of adiponectin, mRNA expressions of AdipoR1 and AdipoR2, and protein expression of APPL1 in myoblasts was observed. These expressions were up-regulated by myogenic differentiation. Up-regulation of adiponectin-related molecules was observed during functional overloading-associated muscle hypertrophy and during the regrowth of unloading-associated atrophied soleus muscle although the physiological role(s) of up-regulation of adiponectin in hypertrophied and regrowing skeletal muscle remains unclear. Therefore, mechanical loading, which is a type of hypertrophic stimuli, might up-regulate adiponectin and its related molecules in skeletal muscle.

## Supporting Information

Figure S1 **Relative mRNA expression levels of glyceraldehyde-3-phosphate dehydrogenase (GAPDH), β-actin, and 18 S rRNA in myoblasts (Mb) and myotubes (Mt).** Mean ± SEM. n = 6.
(TIF)

Figure S2 **Relative mRNA expression levels of glyceraldehyde-3-phosphate dehydrogenase (GAPDH), β-actin, and 18 S rRNA in response to functional overloading.** Cont: untreated control group, OL: functional overloaded group, 1 week and 3 weeks: 1- and 3-week of functional overloading. Mean ± SEM. n = 5/group at each time point.
(TIF)

Figure S3 **Relative mRNA expression levels of glyceraldehyde-3-phosphate dehydrogenase (GAPDH), β-actin, and 18 S rRNA in response to 2 weeks of hindlimb suspension followed by 4 weeks of recovery.** Pre: before hindlimb suspension. R0, R2, and R2: immediately, 2 and 4 weeks of recovery after the suspension, respectively. Mean ± SEM. n = 5/group at each time point.
(TIF)

Figure S4 **Relative protein expression levels of glyceraldehyde-3-phosphate dehydrogenase (GAPDH) and β-actin in myoblasts (Mb) and myotubes (Mt).** Mean ± SEM. n = 6.
(TIF)

Figure S5 **Relative protein expression levels of glyceraldehyde-3-phosphate dehydrogenase (GAPDH) and β-actin in response to functional overloading.** Cont: untreated control group, OL: functional overloaded group, 1 week and 3 weeks: 1- and 3-week of functional overloading. Mean ± SEM. n = 5/group at each time point.
(TIF)

Figure S6 **Relative protein expression levels of glyceraldehyde-3-phosphate dehydrogenase (GAPDH) and β-actin in response to 2 weeks of hindlimb suspension followed by 4 weeks of recovery.** Pre: before hindlimb suspension. R0, R2, and R2: immediately, 2 and 4 weeks of recovery after the suspension, respectively. Mean ± SEM. n = 5/group at each time point.
(TIF)

Figure S7 **Representative images of undifferentiated myoblasts and differentiating myotubes 2, 4, and 7 days after the initiation of differentiation.** Day 2, 4, and 7: 2, 4, and 7 days after the initiation of differentiation
(TIF)
