# Supplementary figures and images for: Up-Regulation of Adiponectin Expression in Antigravitational Soleus Muscle in Response to Unloading Followed by Reloading, and Functional Overloading in Mice
Source: PLoS One. 2013 Dec 6;8(12):e81929. doi: 10.1371/journal.pone.0081929 (PMC3855747; doi:10.1371/journal.pone.0081929)

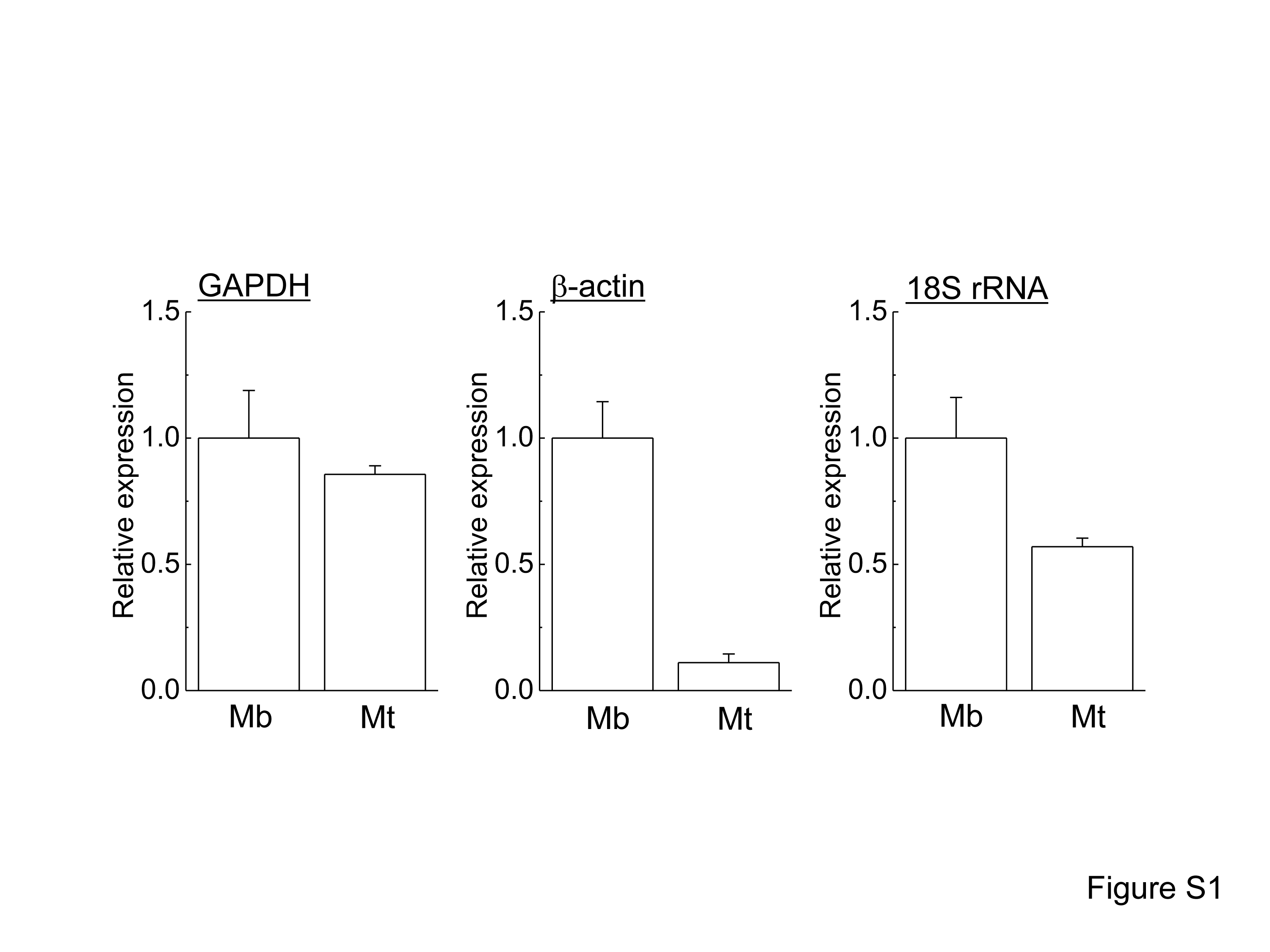

Supplement: Figure S1 — Relative mRNA expression levels of glyceraldehyde-3-phosphate dehydrogenase (GAPDH), β-actin, and 18 S rRNA in myoblasts (Mb) and myotubes (Mt). Mean ± SEM. n = 6. (TIF) [file pone.0081929.s001.tif]

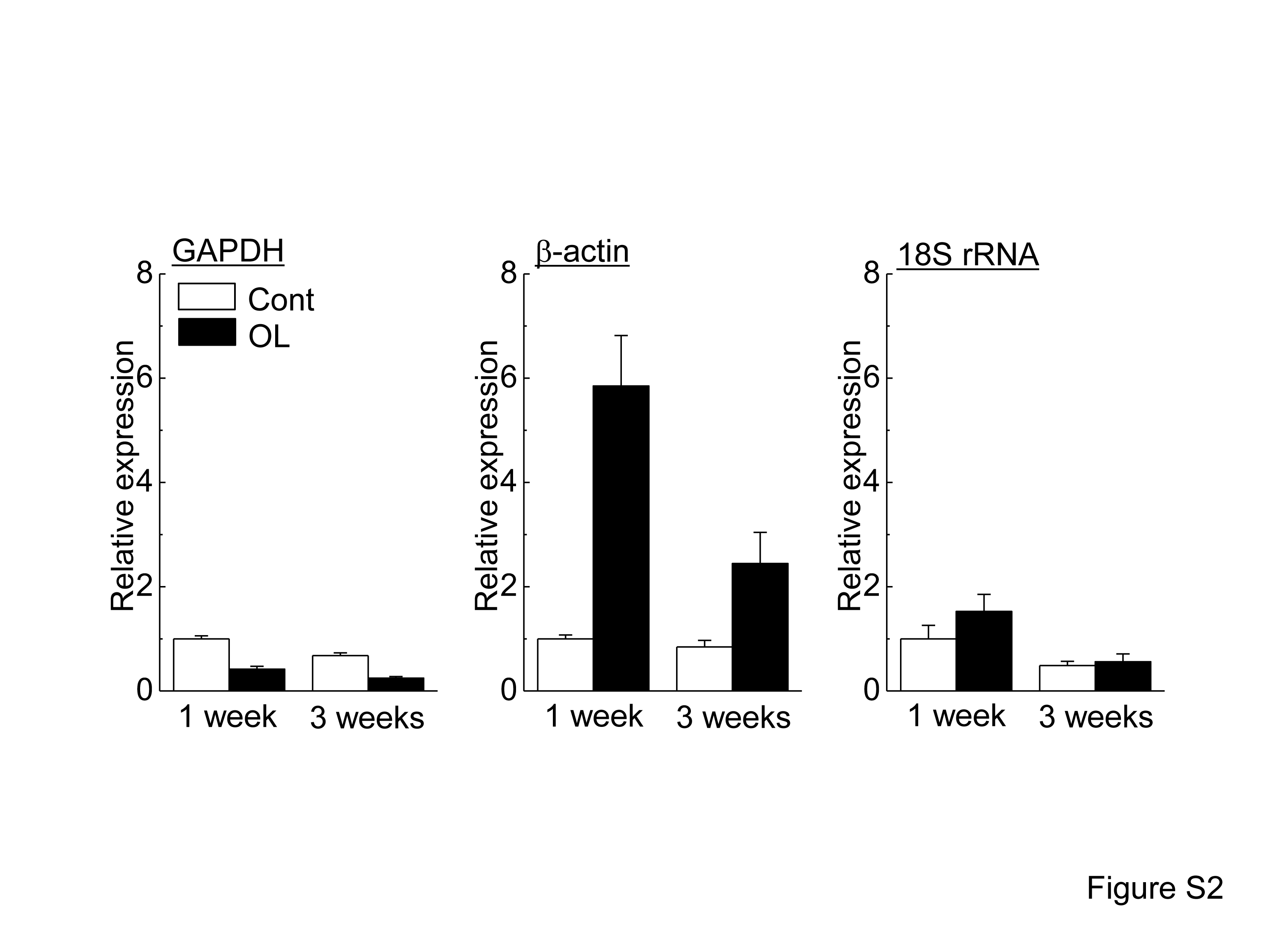

Supplement: Figure S2 — Relative mRNA expression levels of glyceraldehyde-3-phosphate dehydrogenase (GAPDH), β-actin, and 18 S rRNA in response to functional overloading. Cont: untreated control group, OL: functional overloaded group, 1 week and 3 weeks: 1- and 3-week of functional overloading. Mean ± SEM. n = 5/group at each time point. (TIF) [file pone.0081929.s002.tif]

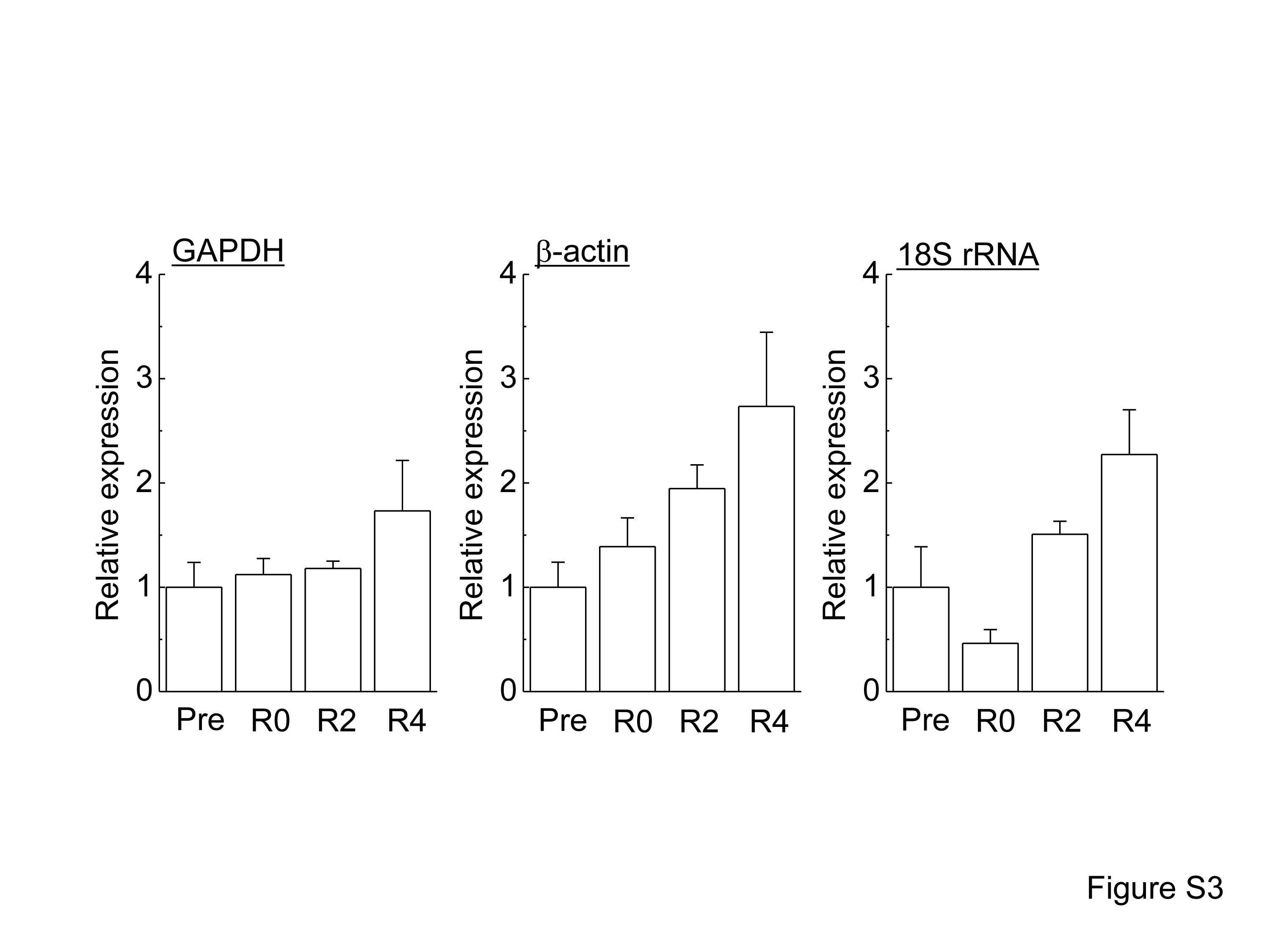

Supplement: Figure S3 — Relative mRNA expression levels of glyceraldehyde-3-phosphate dehydrogenase (GAPDH), β-actin, and 18 S rRNA in response to 2 weeks of hindlimb suspension followed by 4 weeks of recovery. Pre: before hindlimb suspension. R0, R2, and R2: immediately, 2 and 4 weeks of recovery after the suspension, respectively. Mean ± SEM. n = 5/group at each time point. (TIF) [file pone.0081929.s003.tif]

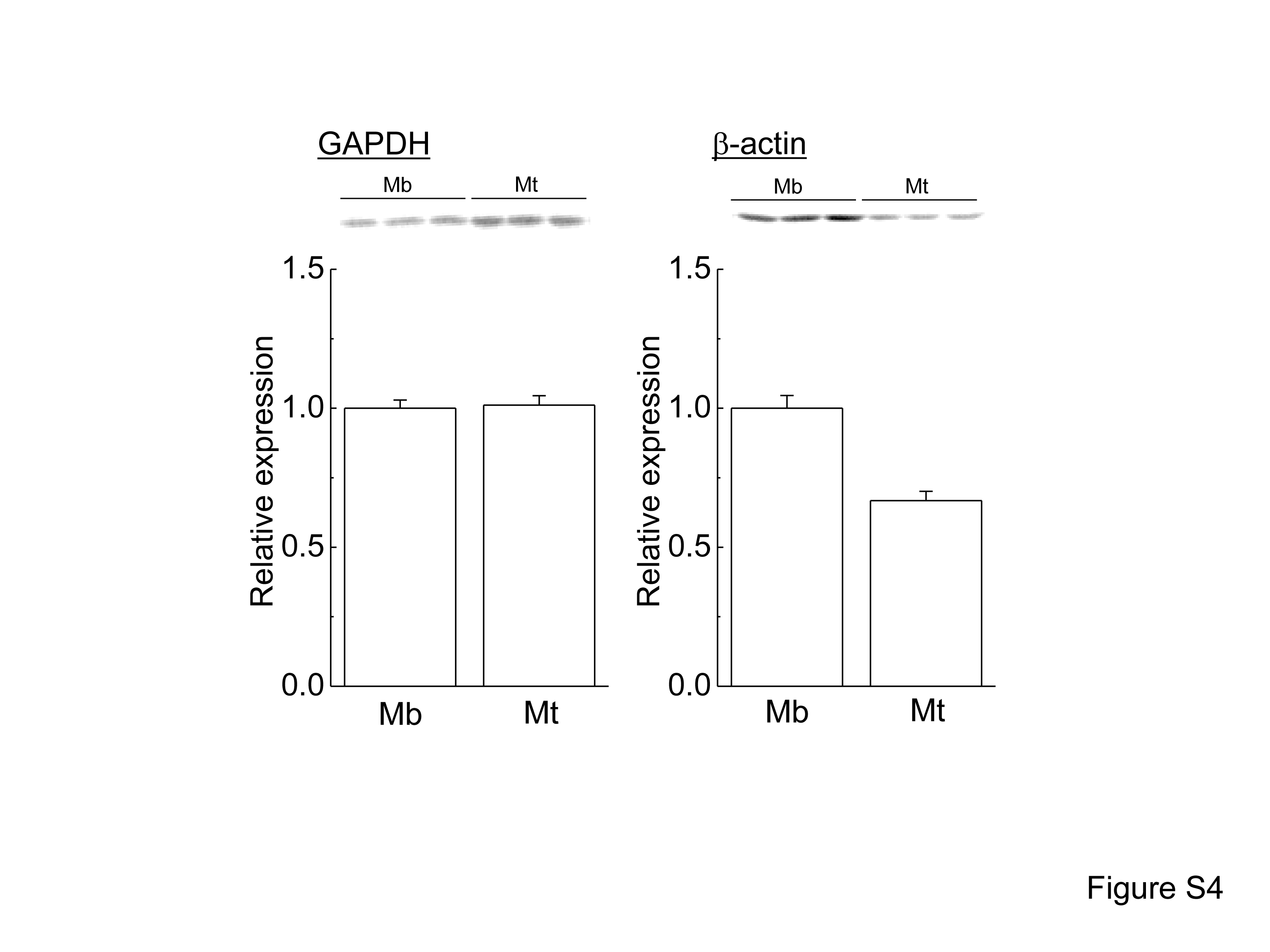

Supplement: Figure S4 — Relative protein expression levels of glyceraldehyde-3-phosphate dehydrogenase (GAPDH) and β-actin in myoblasts (Mb) and myotubes (Mt). Mean ± SEM. n = 6. (TIF) [file pone.0081929.s004.tif]

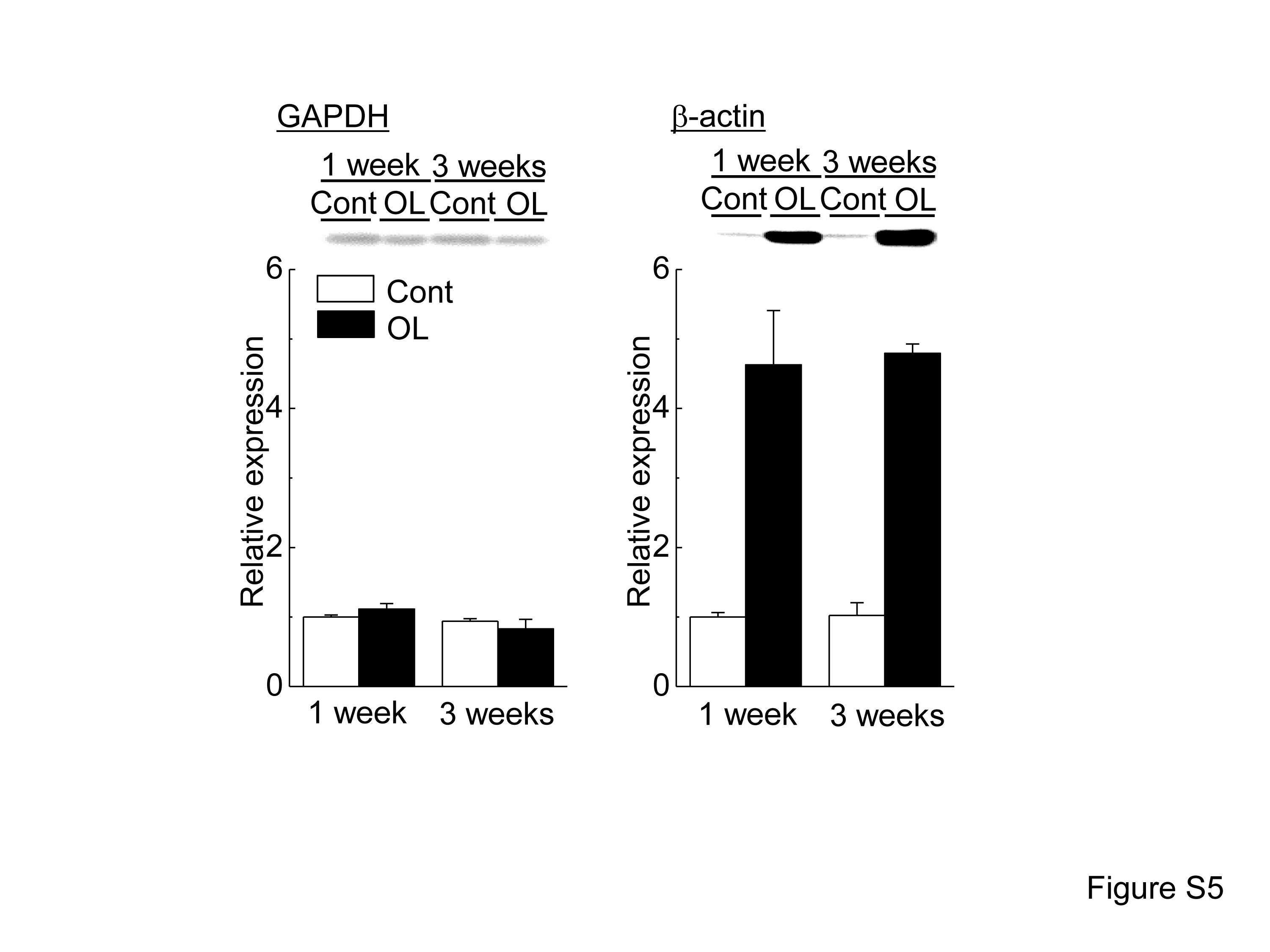

Supplement: Figure S5 — Relative protein expression levels of glyceraldehyde-3-phosphate dehydrogenase (GAPDH) and β-actin in response to functional overloading. Cont: untreated control group, OL: functional overloaded group, 1 week and 3 weeks: 1- and 3-week of functional overloading. Mean ± SEM. n = 5/group at each time point. (TIF) [file pone.0081929.s005.tif]

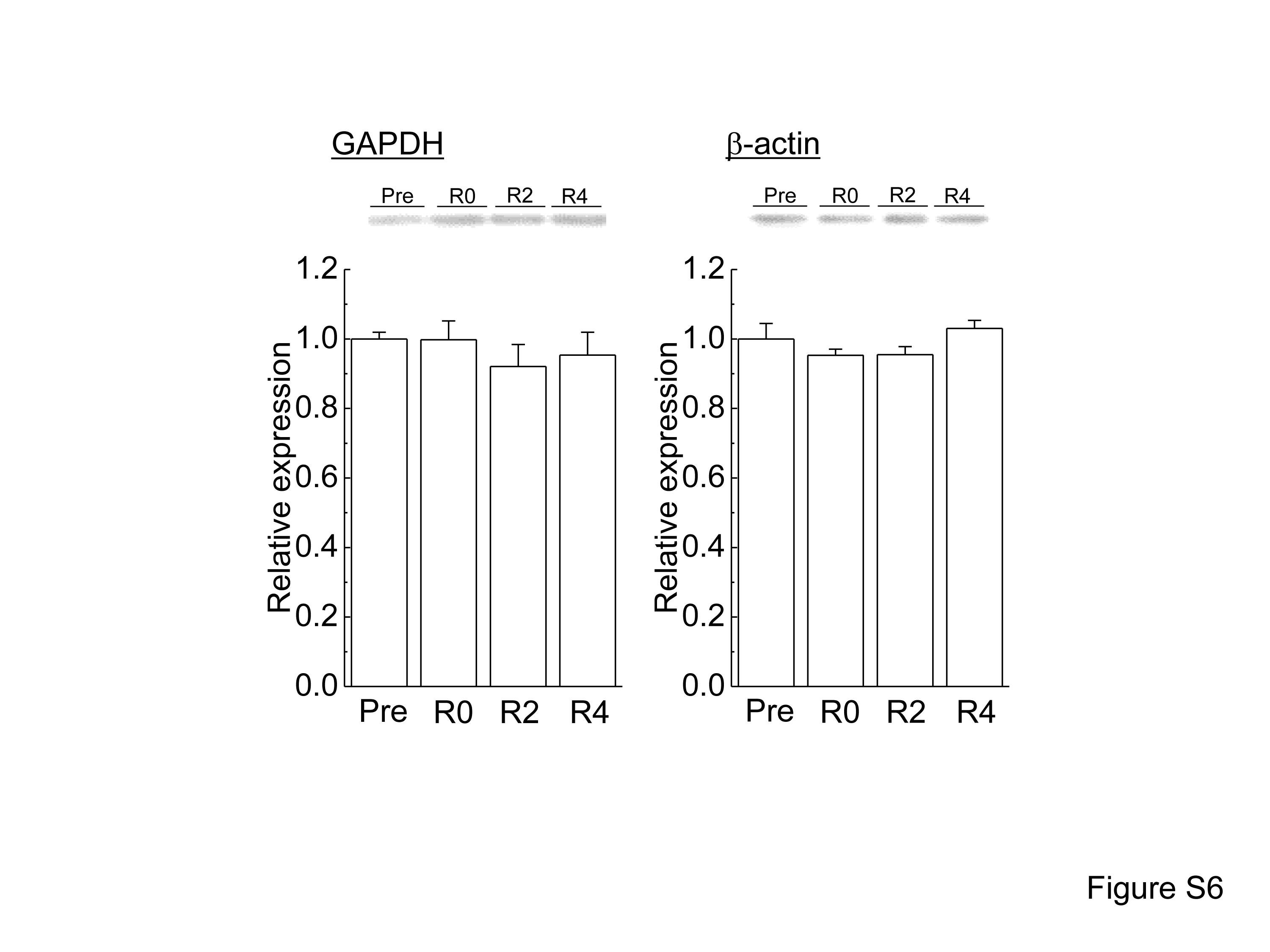

Supplement: Figure S6 — Relative protein expression levels of glyceraldehyde-3-phosphate dehydrogenase (GAPDH) and β-actin in response to 2 weeks of hindlimb suspension followed by 4 weeks of recovery. Pre: before hindlimb suspension. R0, R2, and R2: immediately, 2 and 4 weeks of recovery after the suspension, respectively. Mean ± SEM. n = 5/group at each time point. (TIF) [file pone.0081929.s006.tif]

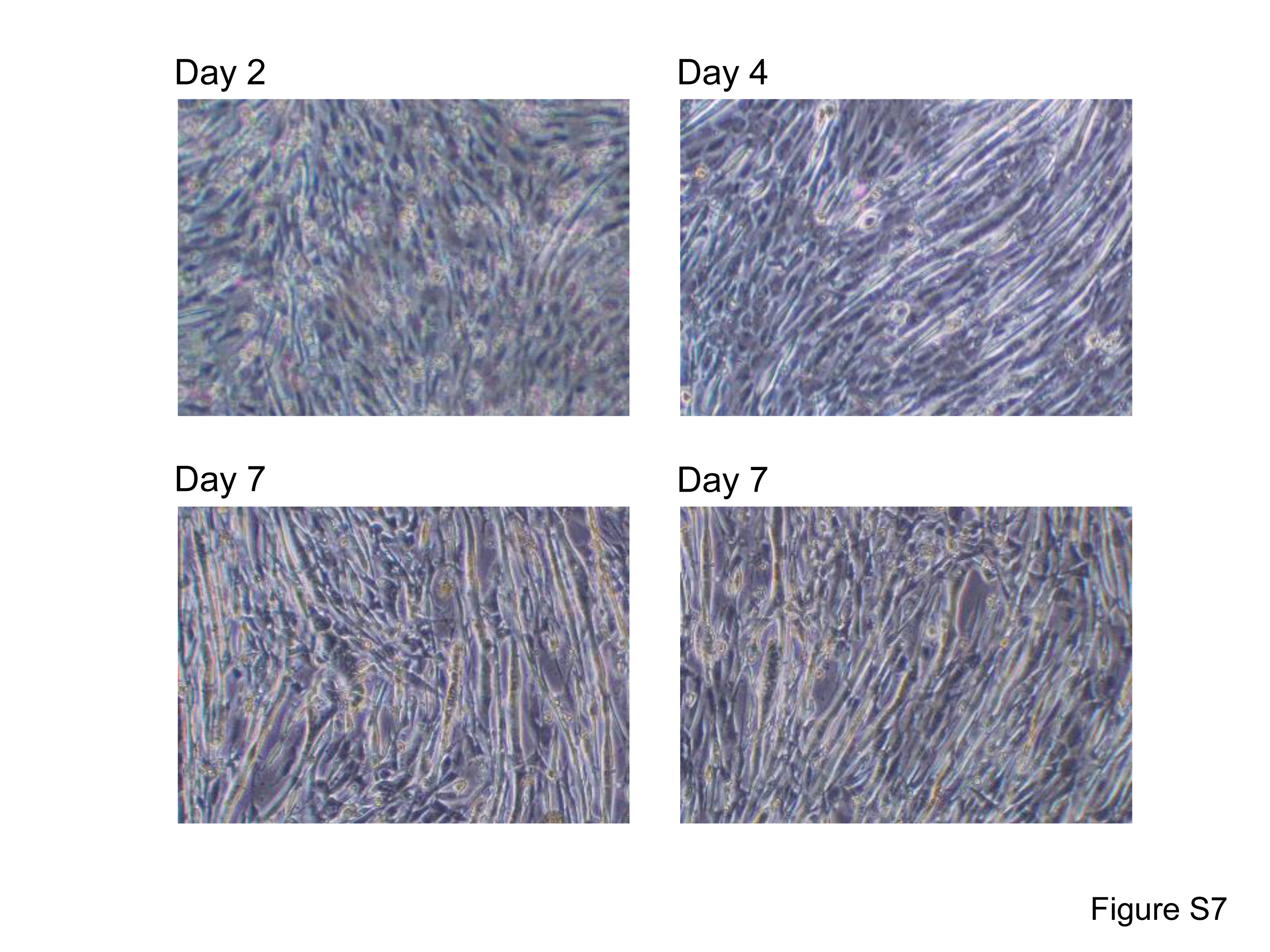

Supplement: Figure S7 — Representative images of undifferentiated myoblasts and differentiating myotubes 2, 4, and 7 days after the initiation of differentiation. Day 2, 4, and 7: 2, 4, and 7 days after the initiation of differentiation (TIF) [file pone.0081929.s007.tif]
